# Supplementary material for: Non-equivalent antigen presenting capabilities of dendritic cells and macrophages in generating brain-infiltrating CD8+ T cell responses
Source: Nat Commun. 2018 Feb 12;9:633. doi: 10.1038/s41467-018-03037-x (PMC5809416; doi:10.1038/s41467-018-03037-x)
Supplement: Supplementary file 1 — Supplementary Information [file 41467_2018_3037_MOESM1_ESM.pdf]

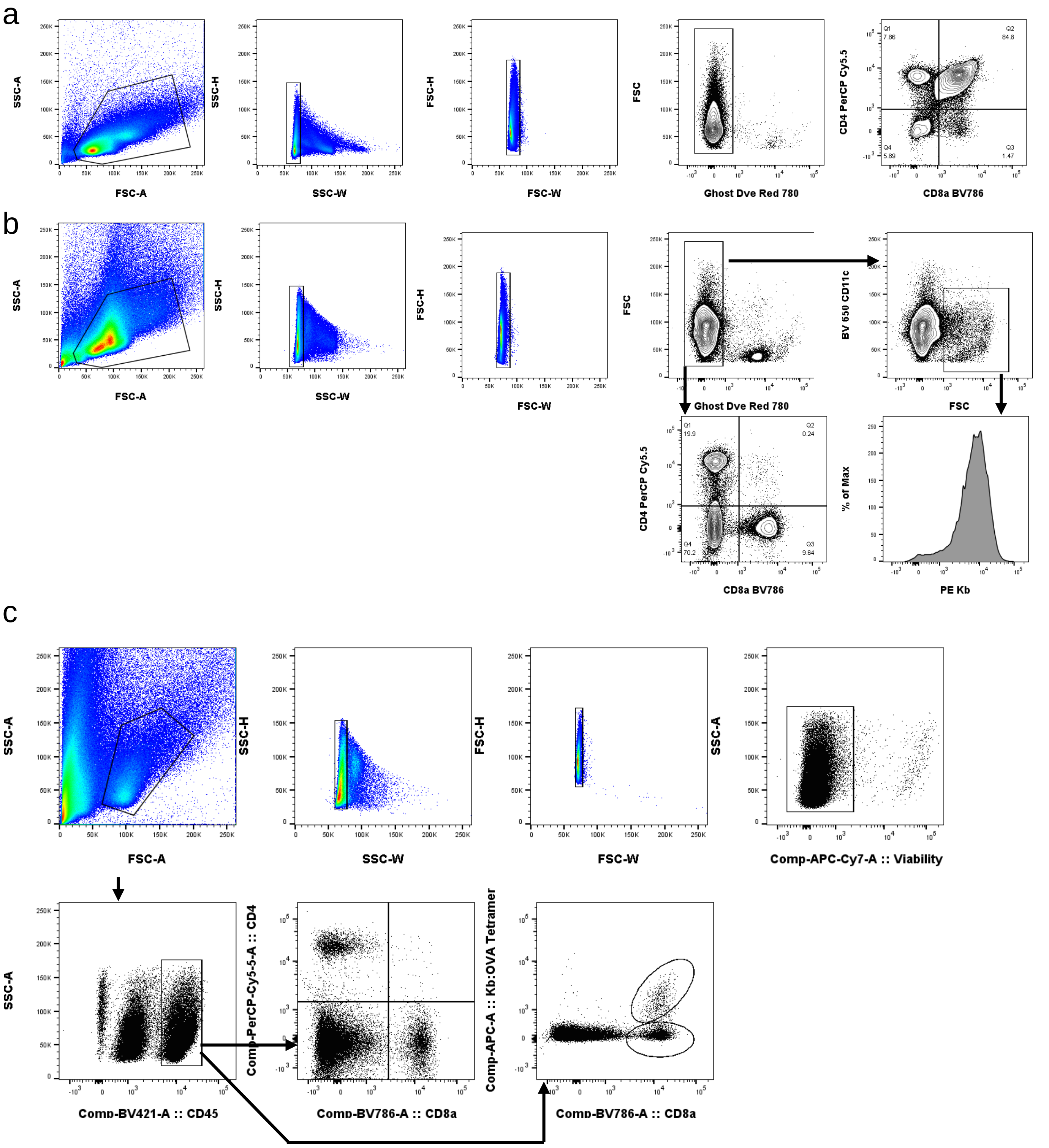

Supplementary Figure 1: Gating strategies. (a) Representative gating for analysis of thymocyte samples (Figure 2, Supplementary Figure 3). (b) Representative gating scheme for analysis of spleen samples (Figures 1, 2, 3). (c) Representative gating scheme for analysis of brain infiltrating lymphocytes (Figures 4, 6, 7, Supplementary Figure 5c).

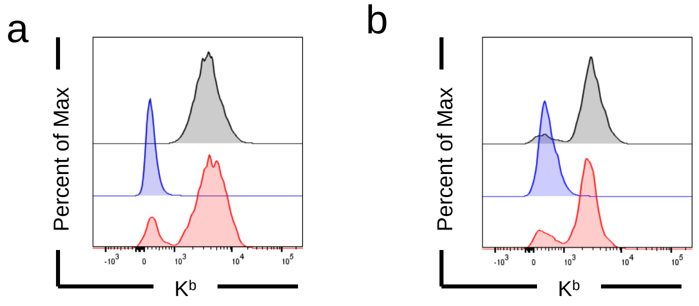

Supplementary Figure 2: Cre-mediated recombination of the H-2K^b transgene is infrequent in microglia during inflammation. Assessment of CD45^{int} CD11c$^+$ microglia (a) 7 days post TMEV-OVA infection and (b) 28 days post GL261 quad cassette inoculation. As expected, cre-negative littermates express high levels of K^b MHC class I in CD11c$^+$ microglia. CMV-cre K^b cKO microglia express little to no K^b. CD11c-cre K^b cKO microglia demonstrate infrequent K^b deletion.

a b

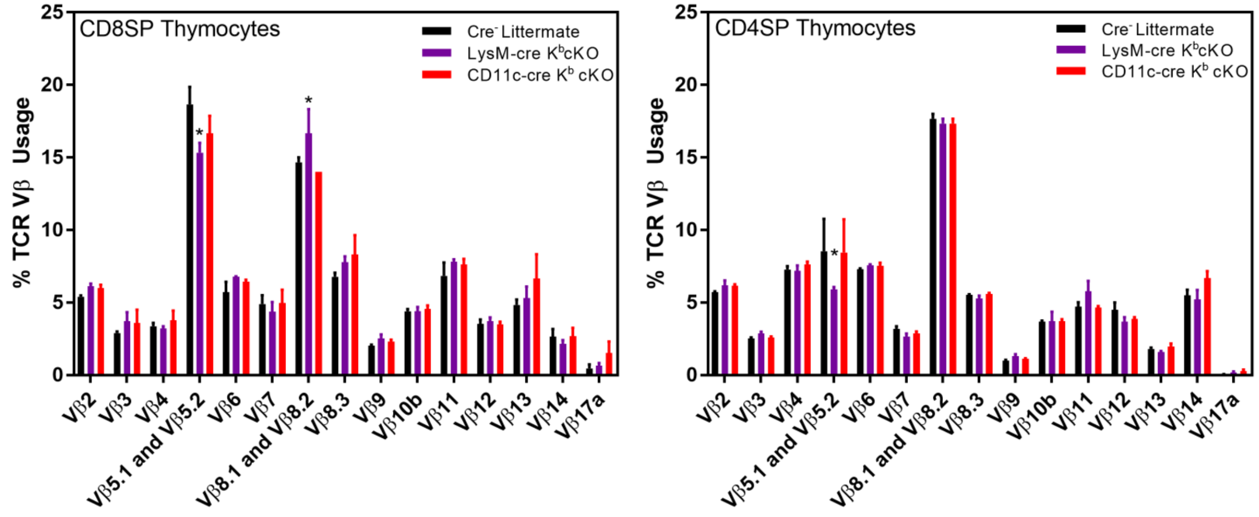

Supplementary Figure 3: Thymic TCR Repertoire is not markedly impacted by cell-specific H-2K^b deletion. Thymocytes were isolated from naïve 6-8 week old animals (N=3 per group) and stained with anti-CD45, anti-CD4, and anti-CD8 antibodies, as well as antibodies specific to each of the T cell receptor Vβ regions listed. Thymocytes at the CD8 single positive (a), and CD4 single positive (b) stages demonstrate very little change to the TCR repertoire as a result of conditional K^b deletion. Data presented as mean with error bars representing standard error of the mean (SEM). * denotes $p<0.05$ by two-way ANOVA with Tukey's correction.

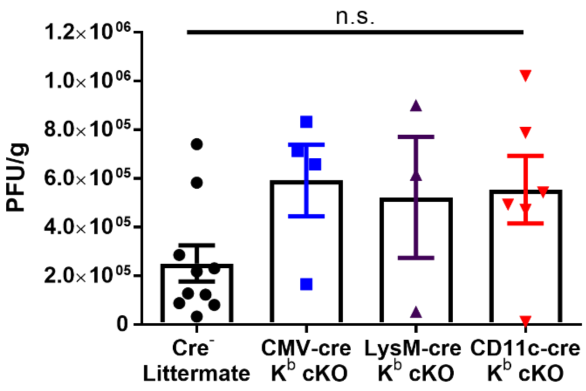

Supplementary Figure 4: Viral Load following TMEV-OVA Infection. Measure of viral load 7 days post infection by plaque assay demonstrates no significant difference in viral load in the CNS and spinal cord of K^b cKO animals (N=10, 4, 3, 6 per group, respectively). Data presented as mean with error bars representing standard error of the mean (SEM). n.s. denotes $p>0.05$ by one-way ANOVA with Holm-Sidak correction.

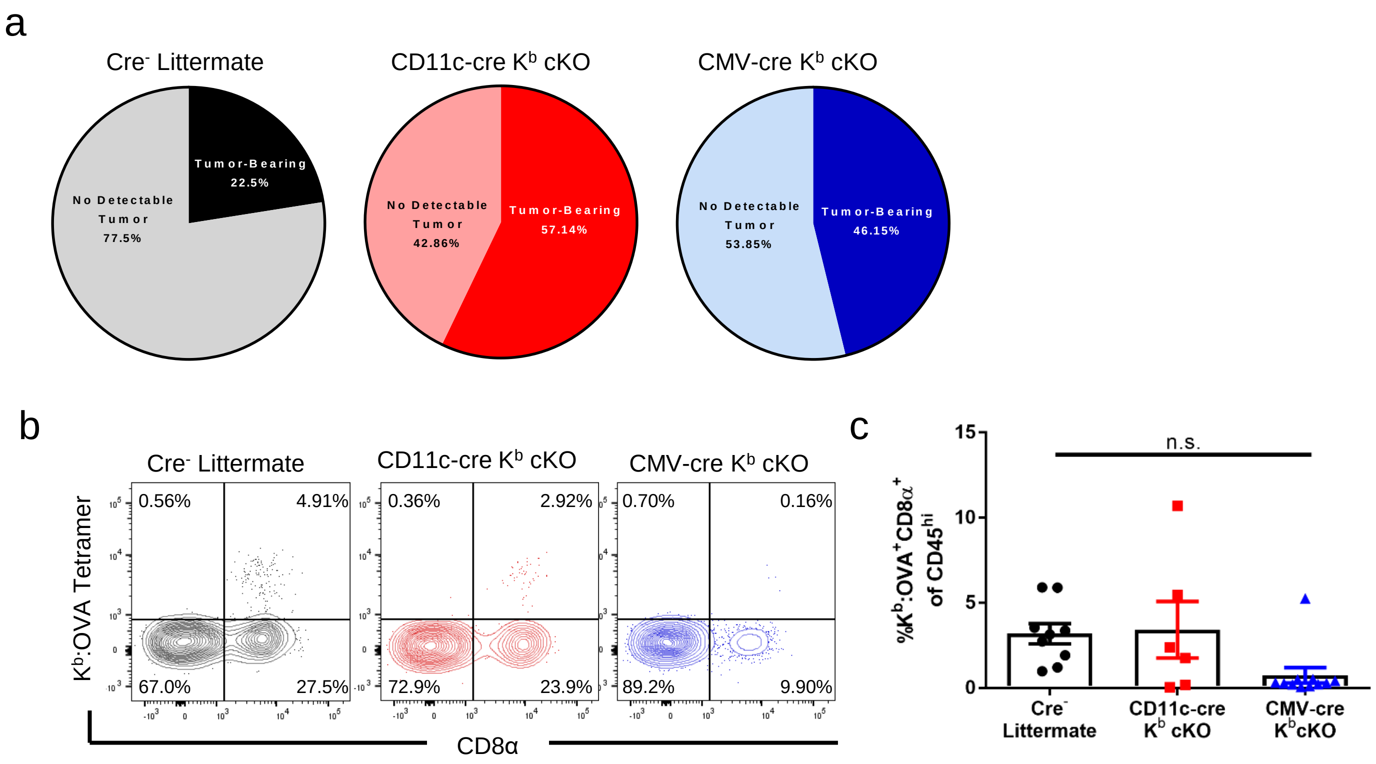

Supplementary Figure 5: The natural response to GL261-quad cassette gliomas is unaffected by cell-specific K^b deletion. (a) Incidence of GL261-quad cassette glioma development. (b) Representatively plots and (c) quantification demonstrate that CD11c-cre K^b cKO animals respond to GL261-gliomas naturally comparably to littermate controls and CMV-cre K^b cKO animals (N=9, 11, 6 per group, respectively. Data presented as mean with error bars representing standard error of the mean (SEM). n.s. denotes $p>0.05$ by one-way ANOVA with Holm-Sidak correction.
